# First French study relative to preconception genetic testing: 1500 general population participants’ opinion

**DOI:** 10.1186/s13023-021-01754-z

**Published:** 2021-03-12

**Authors:** Valérie Bonneau, Mathilde Nizon, Xenia Latypova, Aurélie Gaultier, Eugénie Hoarau, Stéphane Bézieau, Guy Minguet, Mauro Turrini, Maud Jourdain, Bertrand Isidor

**Affiliations:** 1 Département de Médecine Générale, Université de Nantes, 44000 Nantes, France; 2 USR 3491 Maison Des Sciences de L’Homme Ange Guepin, 44000 Nantes, France; 3 Service de Génétique Médicale, CHU Nantes, 9 quai Moncousu, 44093 Nantes Cedex 1, France; 4 Plateforme de Méthodologie Et de Biostatistique, Centre Hospitalier Universitaire de Nantes, Nantes, France; 5 Mines-Nantes School, 44300 Nantes, France; 6 Université de Nantes, Droit et Changement Social UMR 6297, Maison Des Sciences de L’Homme Ange Guépin, Nantes, France

**Keywords:** Preconception genetic testing, Expanded carrier screening, Reproductive medicine

## Abstract

**Background:**

Until very recently, preconception genetic testing was only conducted in particular communities, ethnic groups or families for which an increased risk of genetic disease was identified. To detect in general population a risk for a couple to have a child affected by a rare, recessive or X-linked, genetic disease, carrier screening is proposed in several countries. We aimed to determine the current public opinion relative to this approach in France, using either a printed or web-based questionnaire.

**Results:**

Among the 1568 participants, 91% are favorable to preconception genetic tests and 57% declare to be willing to have the screening if the latter is available. A medical prescription by a family doctor or a gynecologist would be the best way to propose the test for 73%, with a reimbursement from the social security insurance. However, 19% declare not to be willing to use the test because of their ethic or moral convictions, and the fear that the outcome would question the pregnancy. Otherwise, most participants consider that the test is a medical progress despite the risk of an increased medicalization of the pregnancy.

**Conclusion:**

This first study in France highlights a global favorable opinion for the preconception genetic carrier testing under a medical prescription and a reimbursement by social security insurance. Our results emphasize as well the complex concerns underpinned by the use of this screening strategy. Therefore, the ethical issues related to these tests include the risk of eugenic drift mentioned by more than half of the participants.

**Supplementary Information:**

The online version contains supplementary material available at 10.1186/s13023-021-01754-z.

## Introduction

Preconception genetic testing aims to inform of a possible increased risk of having a child with a rare severe genetic condition, providing carrier couples with options for autonomous reproductive choice [1, 2]. This screening strategy is performed on a DNA sample collected from both partners to determine whether they are carriers of deleterious variants in genes associated with recessive or X-linked genetic disorders [3]. Preconception genetic tests can be proposed to allow the couples to prospectively consider relevant reproductive options. If the couple is identified as being at risk to give birth to a child affected by a severe untreatable genetic disease, the options would then be to pursue a standard pregnancy follow–up, to strengthen the monitoring of the pregnancy or to consider prenatal genetic testing. For a subsequent pregnancy, invasive prenatal testing, gamete donation and preimplantation genetic diagnosis procedures will constitute possible choices for the couple. Inversely, a negative result might be associated to an increased confidence in reproductive plans [4].

In France, preconception genetic testing can be proposed for a limited number of genetic disorders, either for relatives of a child with autosomal recessive disease, usually in case of a high frequency of heterozygotes in the general population, or for consanguineous couples. On September 25, 2018, the National Consultative Ethics Committee for Health and Life Sciences (*Comité Consultatif National d'Éthique*) issued a favorable opinion to preconception genetic testing, in the context of the revision of bioethics law. The committee suggested that preconception genetic diagnosis could be offered to everyone of childbearing age who wishes genetic counselling [5]. To date, although multiple initiatives of preconception carrier screening in general population are currently running in Europe, the French general opinion concerning this type of test has never been evaluated.

Definition of target population needs and suitable communication are key elements to a sustainable implementation of a health program [6, 7]. In particular, concerns raised during preconception genetic screening programs set-up might be heavily influenced by population-specific factors. The determination of a target population, the list of tested genetic diseases, the detection and interpretation strategies applied to the identified genetic variants, the access procedure to this test in the context of the existing institutional framework and, more generally, the way to successfully integrate this test into the health care system represent some of the main questions addressed. To collect French general population opinion on the implementation of preconception genetic testing and explore the factors associated with a difference of opinion, we conducted a survey based on an online and printed questionnaire. We questioned the participants on the expected framework and procedures during a preconception test procedure in France.

## Materials and method

Our work is a quantitative, descriptive study carried out using an anonymous poll in general population. The survey ran from 10/11/2017 to 05/03/2018. Basic precisions relative to the aims and modalities of preconception genetic screening were presented to the participants in an invitation letter which accompanied the questionnaire (Additional file 1: Note). The questionnaire, confidential and anonymous, consisted of ten either multiple or single choice questions and was expected to be completed in 5 to 10 min (Additional file 1: Note). A qualitative pre-test phase interrogating 5 individuals has previously been conducted to clarify the questions. Socio-demographic data were collected at the end of the questionnaire. The questionnaire was proposed as a paper document in waiting rooms of medical practices and several places related to the practice of medicine or with social purposes in the region *Loire Atlantique*, or as an online survey. The digital questionnaire was configured via an interface configured by the Sphinx® software (https://sphinx.chu-nantes.fr/v4/s/3dyitq). The participants were recruited via social networks, personal, professional mailing lists and cultural associations. Participants were given the opportunity to receive a feedback note including the outcome of the survey upon request.

Data were analyzed by Epi info™ 3.5 software. We performed a descriptive analysis of socio-demographic characteristics and responses to questions (Tables 1, 2). Qualitative variables were described with the numbers and percentages of each category and quantitative variables with mean and standard deviation. We aimed to determine which variables were associated with being favorable to preconception genetic tests (question 2), by comparing the participants who answered "yes, without any reserve" or "yes, in the context of regulated procedures ", to those who answered "no". Bivariate analyzes were performed using Chi2 or Fisher tests depending on the numbers of participants involved. Variables with a critical probability of less than 0.2 were then integrated into a multivariate model (logistic regression). The variables were then removed one by one from the model, down to the minimization of the AIC (Akaike Information Criterion), to keep only the most informative variables.

**Table 1:** Socio-demographic characteristics of participants

	Number of participants	Percentages
Gender		
Men	358	23
Women	1200	77
Age in years		
[18–28]	360	23
[28–38]	494	32
[38–50]	271	17
[50–65]	335	21
More than 65	90	6
Not specified	18	1
Marital status		
Single	217	14
In a relationship	1232	79
Divorced	84	5
Widow/Widower	17	1
Not specified	18	1
Level of studies		
No degree or certificate of primary education	26	2
Junior college	42	3
Bachelor's degree or equivalent	300	19
Superior short (bachelor + 2)	238	15
Superior long (bachelor > 2)	953	61
Not specified	9	1
Current professional status		
In activity	1122	72
Job search	49	3
In disability	10	1
Retired	164	10
Home	61	4
Student	138	9
Other	15	1
Not specified	9	1
Current professional activity or last profession		
Farmer	10	1
Artisan, merchant and CEO	38	2
Executive and intellectual profession	606	39
Intermediate profession	544	35
Worker	26	2
Employee	164	10
Employee or worker	86	5
Not specified	94	6
Medical professions		
Physician	162	10
Paramedic excluding students	361	23
Social protection status		
Health insurance	1428	91
Universal health cover	47	3
Long term illness	27	2
Other, including:	24	2
Foreign insurance	8	0.5
Independent health insurance	6	0.4
Not specified	29	2
Do you have a child?		
Yes	1055	67
No	499	32
Not specified	14	1
Do you have a parental project?		
Yes	453	29
No	1087	69
Not specified	28	2
Are you or have you ever been confronted, personally or in your entourage, with disability situations?		
Yes	979	62
No	573	37
Not specified	16	1
Do you have a disability?		
Yes	62	4
No	1493	95
Not specified	13	1
Do you have a child with a disability?		
Yes	80	5
No	1468	94
Not specified	20	1
Does anyone in your family have a disability?		
Yes	511	33
No	1038	66
Not specified	19	1
You or your entourage, have you already been concerned by medically assisted procreation?		
Yes	495	32
No	1059	67
Not specified	14	1
You or your entourage, have you ever been involved in a genetic study?		
Yes	437	28
No	1121	71
Not specified	10	1

**Table 2:** Bivariate analysis of question 1 and 2

	Question 1: Are you aware of this type of test?				
	Yes		No		Chi 2 test
	Number of participants	Percentages	Number of participants	Percentages	
Physicians	58	35	110	65	*p* = 0.09
Paramedics	71	32	151	68	
Not medical or paramedical	323	27	853	73	

	Question 2: How would you like to be informed about the existence of this test?				
	Women		Men		Chi 2 test
	Number of participants	Percentages	Number of participants	Percentages	
By the family doctor	726	46	246	16	*p* < 0.001
By the gynecologist	822	52	182	12	

## Results

### Descriptive analysis of the population

**Fig. 1:**
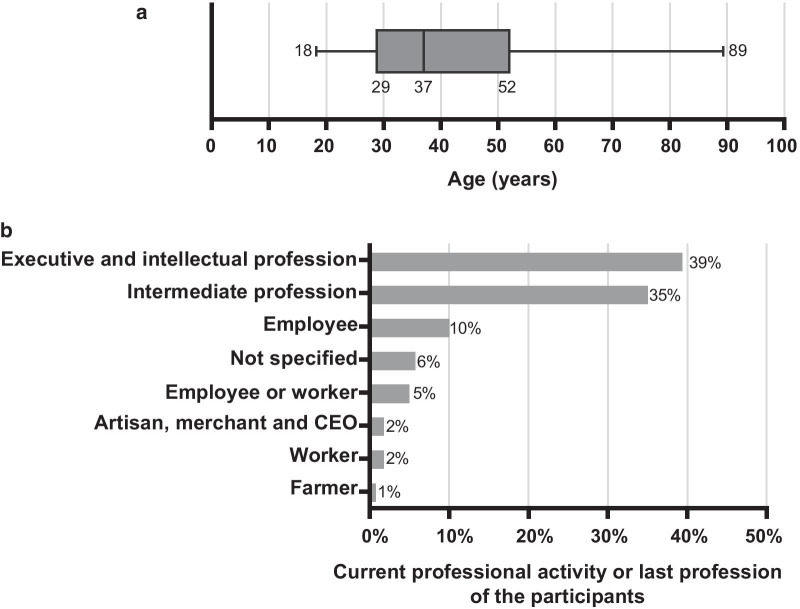
**a** Box diagram of the ages of the participants. The diagram shows the median observation and values falling outside the 25th and 75th percentile are plotted as outliers. **b** Distribution of the sample by professional category

One thousand five hundred sixty-eight individuals answered the questionnaire. Our sample includes 77% women and 23% men (Table 1). The average age is 40 years (standard deviation = 14.5) and the population aged 18–37 represents 50% of our sample (Fig. 1a). All socio-professional categories are represented (Table 1, Additional file 2: Table S1), with a prevalence of senior managers and higher intellectual professions (39%) (Fig. 1b). Graduates represent 76% of our sample (Table 1). One hundred sixty-two (10%) are physicians and 361 (23%) are paramedics. Seventy-nine percent of participants are in a relationship, 67% have at least one child and 29% have a parental project. Sixty-two percent of participants have previously been confronted to a disability, 33% have a relative with a disability and 4% have a disability. Thirty two percent have already been concerned by the medically assisted procreation and 28% by a genetic study (Table 1). Two hundred participants completed the paper-based questionnaire collected in medical and paramedical practices.

### Test availability in France

**Fig. 2:**
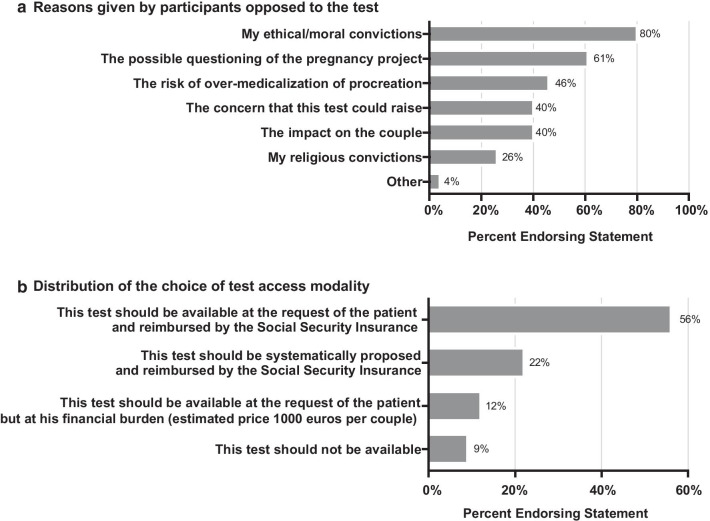
**a** Reasons given by participants opposed to the test. **b** Distribution of the choice of test access modality

Ninety-one percent of respondents are in favor of an access to a preconception genetic test in France, for 61% in the context of regulated procedures, and for 30% outside this framework. Nine percent of the participants declare to be opposed to the test. The two main arguments advanced by the opponents of the test are ethical and moral convictions for 80% of the participants, and the possible questioning of the pregnancy project for 61% of the participants (Fig. 2a). A majority (73%) would be favorable to a test performed after a medical prescription, of which 49% favorable to a test accessible to everyone and 24% favorable to the test proposed according to the medical history. Twenty-one percent of participants believe that the test should be accessible to everyone, either with or without a medical prescription. A majority (78%) of participants believe that the test should be reimbursed by the national health insurance, of which most (56%) favor a test proposed at the request of the patient and 22% favor a test systematically proposed to the patient (Fig. 2b). For 52% of the participants, this test should be offered to any couple with a parental project, for 51% of them it should be proposed to couples with relatives with a serious illness and for 49% to couples with a child affected by a serious illness (Additional file 2: Table S2).

### Intention to perform the test

**Fig. 3:**
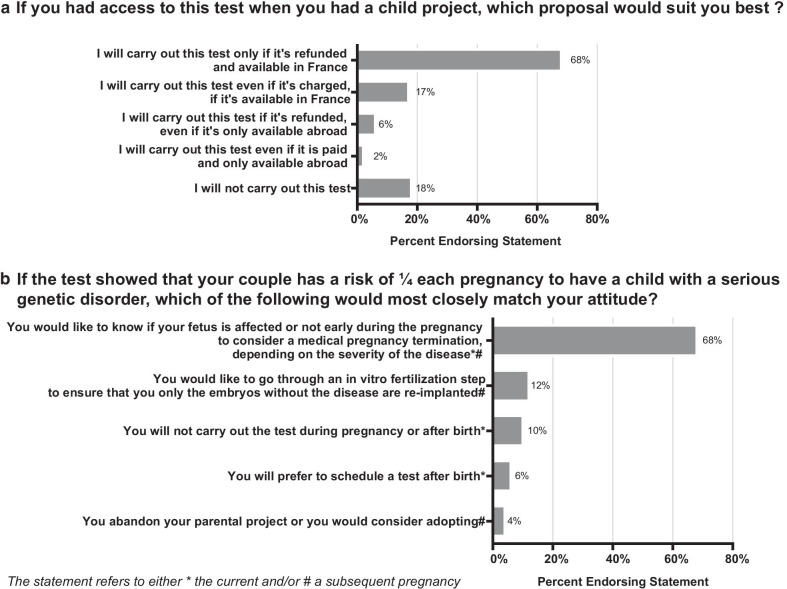
**a** Distribution of answers to question 5: If you had access to this test when you had a child project, which proposal would suit you best? **b** Distribution of answers to question 9: If the test showed that your couple has a risk of ¼ each pregnancy to have a child with a serious genetic disorder, which of the following would most closely match your attitude?

Sixty-eight percent of the participants would carry out the test if it was available and refunded in France. Fifty-seven percent would like to carry out the test as part of a parental project. Seventeen percent would perform the test, even if they would have to pay an estimated price of 1000 euros per couple. Moreover, 2% would be willing to carry out the test abroad if it was not available in France (Fig. 3). Finally, 18% of the participants would not wish to carry out the test.

The main reasons put forward by the participants not willing to be tested were the ethical and moral convictions (70%), the possible questioning of the pregnancy project (50%) and the anxiety that this test could generate (56%). If the test showed for the couple a 25% risk of having a child with a serious illness, 68% of the participants would undergo a prenatal diagnosis procedure and consider a medical termination of pregnancy depending on the outcome, 12% would choose to go through an in vitro fertilization with preimplantation genetic diagnosis and 4% would abandon their plans for pregnancy. Finally, 10% of participants would not perform a genetic test to inform the status of the child, before or after a child birth (Fig. 3b).

### Way of being informed about the existence of the test

The majority of participants (71%) had never been aware of preconception genetic tests of test before this study. Unexpectedly, being a physician or a paramedical professional is not significantly associated with a difference in the knowledge relative to the existence of the test (*p* = 0.09). About 2/3 of participants would like to be informed of the existence of the test by their family doctor and/or their gynecologist, although 19% would like to be informed by an information campaign issued by the Ministry of Health. Men are more favorable to information provided by the family doctor, while women would prefer information provided by a gynecologist (*p* < 0.001).

### General opinion on the access to a preconception carrier screening in France

**Fig. 4:**
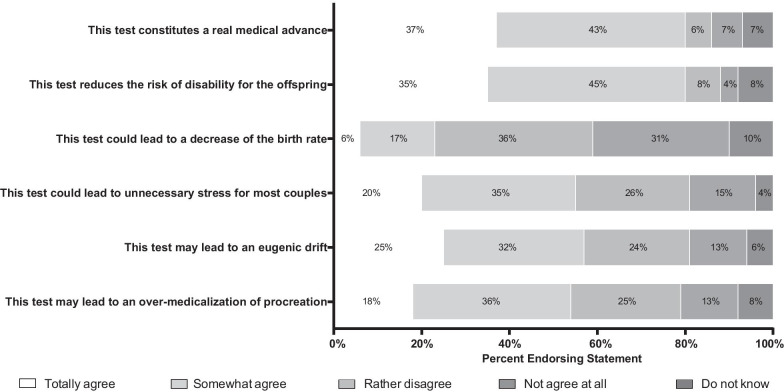
Distribution of answers to question 7: Do you agree with the following proposals?

Eighty percent of participants believe that this test is a real medical advance, reducing the risk of disability. Nevertheless, 57% of the participants believe that the test could lead to eugenic practices, 54% believe that it can lead to over-medicalization of procreation and 55% consider that this test could cause unnecessary stress for most couples (Fig. 4).

To search for a possible statistical association between the socio-demographic data and the mention to be favorable to the test in France, we performed a multivariate analysis and underlined the variables having a statistically significant influence on the answers. An interim analysis was performed. The results of the multivariate analysis are presented in Table 3. Being in favor of preconception genetic tests in France is not statistically significantly associated with gender, socio-economic categories, marital status or personal disability status. The older participants were more in favor of the test. Indeed, 50–65 year-old participants are five times more likely to be in favor of the test than 18–25 year-olds (OR 5.25, CI 1.93–14.98, *p* = 0.001). Medical and paramedical professions are more likely to be in favor of the test (OR 2.87, CI 1.43–6.43, *p* = 0.005). Individuals who had previously been involved in a genetic study were almost twice as likely to be in favor of the test as those not involved in a genetic study (OR 1.83, CI 1.19–2.87, *p* = 0.007). Long-term graduates are three times more likely to have an unfavorable opinion about the test than subjects with no higher education and graduates (OR 1/3.7, CI 0.13–0.51, *p* < 0.001). Short-term graduates are twice as likely to be in favor of the test as participants who did not carry out higher education studies (OR 1/2.6, CI 0.17–0.84, *p* = 0.019). Participants with at least one child are five times more likely to be in favor of the test access than participants without children (OR 1/5, CI 0.11–0.33, *p* < 0.001). Strikingly, participants having a child project are twice more likely to have an unfavorable opinion on the test (OR 1/2.6, CI 0.24 -0.60, *p* < 0.001) and participants with a relative affected by a disability are twice as likely to have an unfavorable opinion on the test (OR 1/2, CI 0.34–0.74, *p* < 0.001).

**Table 3:** Multivariate analysis: In general, would you be in favor of access to this type of test in France?

	Model after variable selection by Akaike information criterion			
	Odds ratio	Confidence interval		*P*
Age				
[18, 25]	1	1	1	Reference
[25, 35]	1.30	0.63	2.66	0.467
[35, 50]	1.96	0.86	4.42	0.105
[50, 65]	5.25	1.93	14.98	0.001
[+65]	13.15	2.30	250.25	0.017
Level of studies				
Bachelor degree or less	1	1	1	Reference
Higher short	1/2.6	0.17	0.84	0.019
Higher long	1/3.7	0.13	0.51	0.0001
Profession				
Not medical	1	1	1	Reference
Medical	2.87	1.43	6.43	0.005
Paramedical	1.92	1.11	3.48	0.025
Do you have a child?				
No	1	1	1	Reference
Yes	1/5	0.11	0.33	1.17E-08
Do you have a parental project?				
No	1	1	1	Reference
Yes	1/2.6	0.24	0.60	4.38E-05
Are you affected by a disability?				
No	1	1	1	Reference
Yes	4.57	0.94	82.47	0.141
Does anyone in your family have a disability?				
No	1	1	1	Reference
Yes	0.50	0.34	0.74	5.53E-04
Have you or anyone in your environment been previously involved in a genetic study?				
No	1	1	1	Reference
Yes	1.83	1.19	2.87	0.007

## Discussion

### Representativeness and generalizability

This is a first-time study in France involving a large sample size. Women, participants under the age of 65, higher intellectual professions, graduates of long-term higher education and physicians are overrepresented in our sample (Table 1, Additional file 2: Table S1). Thus, ten percent of the participants were physicians, comparing to 0.3% in the general population in 2018 (Table 1, Additional file 2: Table S1). These differences can be explained by the access to the questionnaire, which was proposed in waiting rooms of medical practices. Men are frequently underrepresented in health surveys, and are less likely to participate in genetics research [8]. Women were also overrepresented in previous studies dealing with preconception genetic tests [9]. Because access to the internet is a known barrier to participation to genomic research, we proposed both internet and paper-based surveys. However, individuals who did not attend medical or paramedical follow-up could not be reached and participate in the survey because of our questionnaire distribution system. Similarly, participation rate to studies focusing on personal genomics has previously been shown to be lower in individuals living in a lower education neighborhood [8]. Overall, our sample characteristics are comparable to other studies dealing with genomic medicine and in particular preconception genetic tests and our study collects the opinions from more than 1500 participants.

### A global opinion mostly favorable to the test

In our study, most participants declare to be favorable to the access to this type of test in France (91%). Nevertheless, 57% only would consider using this type of test and 19% would expressly decline to use it. These data are close to the results of two previous European surveys. The first survey was conducted in 2014 in Belgian population [10]. The inclusion criteria were to speak Dutch fluently and to be over 16 years old. One thousand one hundred eighty–two participants answered the questionnaire. This survey reports that 53.8% participants expressed their willingness to perform preconception genetic screening for recessive diseases and 60.7% would be interested in prenatal genetic screening. The second survey, conducted in the Netherlands in 2016, concerned the preconception genetic testing for 50 severe diseases [11]. This study reported that 56% of all participants would be interested in a preconception test for serious, incurable and early-onset diseases. In this study, 34% of the participants would carry out the test, if the latter were free. The population analyzed in this study included men and women living as a couple and of childbearing age, which might explain part of the differences with our data, collected without restriction on age and marital status. Overall, our data are consistent with the outcomes of these two studies, and suggest that the opinion of the general population in France is globally positive.

More than 70% of participants consider this test as a medical advance that would reduce the risk of disability for the offspring. In our study, the outcome of preconception genetic testing would prospectively influence couples’ reproductive choices, which is among the main aims of expanded carrier screening strategies [1]. If the test showed that the couple had a 25% risk of having a child with a serious genetic disorder, 67% of the participants would then undergo a prenatal diagnosis and 12% would opt for a preimplantation genetic diagnosis by going through an in vitro fertilization process. The main arguments advanced by the participants in favor of the test during the 2016 Dutch survey were the possibility to preserve their child from a severe hereditary disease (39%) and to avoid to have a child with a severe hereditary disease (14%) [11]. A test proposed to the general population would reduce the risk of inequitable access to the test, and the risk of stigmatization of certain ethnic groups [12]. In 2016, in Denmark, during a study conducted in the Jewish community, 53.8% of the participants were in favor of a test proposed to the general population for reasons of risk of stigmatization and the difficulty of identifying subjects at risk in populations experiencing ethnic mixing [13]. In 2006, during the set up in general population of the screening program for cystic fibrosis in Australia, a study reports the emotional experiences and choices of 10 couples identified as carriers of a deleterious variant, between 2006 and 2010 in the city of Victoria. Couples who were expecting a child at the time of the screening decided to undergo a prenatal screening. Two couples informed that their fetus was affected by cystic fibrosis decided to terminate the pregnancy. All couples involved in the study have modified their parental project [14].

### Opponents to the preconception genetic tests: advanced arguments

The secondary objective of our study was to identify socio-demographic criteria associated with the participants' opinion regarding the access to the test in France. The multivariate analysis of our second question (“Would you be in favor of an access to this type of test in France?”, Additional file 1: Note) showed that having a child, a relative with a disability, or having a degree in either a high or a high school education, is significantly associated with an unfavorable opinion to the access to the test in France. Moreover, individuals with a parenthood project, who are therefore the most directly concerned by the test, are more frequently unfavorable to access to the test than those with no conception project. This trend can possibly be explained by the fear of a possible questioning of the pregnancy project, argument advanced by 61% of the participants unfavorable to the test (Fig. 2a). A qualitative study designed to explore the reasons for this negative opinion on preconception genetic tests would be necessary to yield explanatory elements. Contrariwise, being older than 50, working in the medical or paramedical sector or having previously been involved in a genetic study is statistically significantly associated with an opinion more favorable to access to the test.

To understand the factors associated with an unfavorable opinion on preconception genetics tests, we asked the participants what were for them the reasons against the use of these tests. The participants opposed to the test in France (9%) put mainly forward arguments related to their ethical and moral convictions, a possible questioning of the project of pregnancy and the anxiety that this test could generate (Fig. 2a). Ethical issues previously associated with preconception genetic tests previously include the fear of discrimination and stigmatization in case of a positive test [15, 16]. Moreover, the risk of a shift to eugenic practices is a fear reported by the majority of the sample surveyed (57%), as well as the fear of a risk of unnecessary stress for the majority of couples (55%). Women's health professionals were interviewed during a conference in an American study published in 2012 [17]. The fears associated with this test reported in this study included the concerns about results' confidentiality (40%), the increase in health insurance contributions (37%), and the fear of being discriminated if they were identified as carriers of a deleterious variant (31%) [17]. Additional healthcare costs, anxiety and psycho-social harms from learning about carrier risks have also been mentioned as potential risks associated with preconception genetic testing procedures [18].

### A test prescribed by a medical doctor and reimbursed: an organized, reliable healthcare framework

Our data are in favor of a large tendency favorable to the screening in the context of the test reimbursement by national health insurance (78%) and regulation through prescription by a health professional. Access to genetic tests is currently unauthorised without a medical prescription, in France [19]. Several studies report the necessity of a medical supervision of preconception genetic screening, to inform the parents correctly in case of positive results, and to avoid the confusion relative to the nature of the test, in particular between the preconception test and the prenatal screening test for Down Syndrome [20, 21]. A medical prescription would thereby facilitate the accompaniment of couples, as well as the delivery of information related to the test. In our study, the majority of the subjects interviewed are in favor of a test proposed by the gynecologist or a general practitioner. Sixty-four percent of participants would like an information provided by their general practitioner, similarly to the Dutch study's conclusions, in which 44% of participants would prefer to have a test proposed by their general practitioner [11].

Cost influences test acceptance: in our data, less than 20% of participants would be willing to pay for the screening. These results can notably be explained by the announced price of 1000 Euros in our questionnaire. In the study conducted in Dutch population, 58% of the participants would consider paying for preconception genetic testing [9]. The willingness to pay for genome sequencing carrier screening evaluated in 2018 during the the NextGen study, as part of the Clinical Sequencing Exploratory Research (CSER) consortium [22]. The maximum amounts that participants were willing to pay were related to income, and participants’ highest willingness to pay level has been estimated to $21–100 and $101–300 for female and male partners, respectively. We can assume that a paid test would be a barrier to its achievement and prescription [23]. In his opinion issued on September 25, 2018, the french National Consultative Ethics Committee for Health and Life Sciences proposed that preconception genetic diagnosis could be covered by the Social Security Insurance [5]. That was also the condition for the acceptance of the test for most of the participants in our study (Fig. 3a).

### Need for further reliable information of health professionals involved

General practitioners are particularly concerned with preconception care and pregnancy. In view of our results, the population analyzed in our study would like to be informed by the family doctor on the existence of preconception genetic tests. Whatever their sources of information, these physicians must have sufficient and trustworthy knowledge to guide couples. They should also be prepared to the difficulties associated with the interpretation and communication of test results, which have already been addressed by focus groups of geneticists [24]. The information and training of health professionals on genetic preconception tests would be the challenges of these new tests. According to our data, 65% of physicians were unaware of this type of test in France, and there was no significant difference in terms of knowledge with the other participants. Practitioners should be sufficiently informed to properly guide the couple on the risks and benefits of preconception genetic testing and direct-to-consumer testing [25]. Offering couple-based expanded carrier screening through general practitioners has been shown to be feasible and general practitioners are considered as suitable providers for a preconception genetic tests [26].

## Conclusion

Our study is the first survey of public opinion of the general population in France raising the question relative to the access of preconception genetic tests. We show an overall favorable opinion to the implementation of preconception genetic tests in France. Unexpectedly, participants with a parental project tend to have a less favorable opinion about this test. The main arguments advanced by the non-favorable participants are the ethical and moral convictions, as well as the possible questioning of the pregnancy project. Although the vast majority considers this test as a real medical advance to reduce the risk of disability for offspring, half of participants also mentioned a possible risk of shift to eugenic practices and over-medicalization of procreation. The majority of participants consider that the test should be performed based on a medical prescription, supported by national health insurance, and accompanied by information delivered by health professionals. To facilitate health care of the individuals concerned by this test is one of the challenges of the implementation of expanded preconception screening. Future parents must be informed during the process, before the prescription of this analysis. Genetic professionals and several primary care health professionals will be impacted (obstetrician gynecologists, midwives, general practitioners). At this time it’s not sure these last ones are willing to. Gynecologists might feel uncomfortable with genetics results, probably the same for general practitioners. Whether they will be involved in this, they will need adequate resources to properly guide future parents. At this time, we believe that most participants who would have at least a molecular variant identified would then refer to a genetic professional. This would probably need a new organization for genetic counselling in France. A complementary study of first-line health professionals (obstetrician gynecologists, midwives, general practitioners) would be necessary to explore their opinions on the test, their views of their roles in informing couples, and their expectations regarding medical training to preconception genetic tests.

## Supplementary Information

**Additional file 1: **Notes.

**Additional file 2: Table S1**. Summary table of INSEE French demographic data. **Table S2**. Univariate analysis of questionnaire responses.

## Data Availability

The raw data used and/or analyzed during the study are available from the corresponding author upon reasonable request.
