# Supplementary material for: First French study relative to preconception genetic testing: 1500 general population participants’ opinion
Source: Orphanet J Rare Dis. 2021 Mar 12;16:130. doi: 10.1186/s13023-021-01754-z (PMC7955630; doi:10.1186/s13023-021-01754-z)
Supplement: Supplementary file 1 — Additional file 1: Notes. [file 13023_2021_1754_MOESM1_ESM.docx]

**Supplemental note:**

**Study relative to preconception genetic tests**

Dear Sir/Madam,

Technological advances continuously enable new opportunities to identify a growing set of genetic disorders. A new test giving the opportunity to determine the risk for a couple to have a kid affected by a severe genetic disorder (as cystic fibrosis, myopathies, or intellectual disability) with a parental blood sample will be available soon. Please note that these disorders can occur without any known genetic disease in the patient’s relatives.

We are interested in your opinion on this test.

Our study aims to collect the opinion of any person having reached the age of majority, living in France, although these tests are only available abroad. For that we need a few minutes of your time (5 to 10 minutes) to answer to this questionnaire, which is confidential and anonymous.

To access the questionnaire:

Scan the QR code with your smartphone or your tablet,

Go to the following URL: <https://sphinx.chu-nantes.fr/v4/s/3dyitq>.

Let us know by e-mail if you cannot access the survey with this link to receive a paper or digital version of the questionnaire.

**We would be grateful if you could you share the survey within your contacts and relatives.**

You have the possibility to give us your contact information, whether you wish to be informed of the outcome of this study.

**Study relative to preconception genetic tests**

Technological advances continuously enable new opportunities to identify a growing set of genetic disorders. A new test giving the possibility to determine the risk for a couple to have a child affected by a severe genetic disorder (as cystic fibrosis, myopathies, intellectual disability) through the analysis of a parental blood sample might be available soon. Please note that these disorders can occur without any known genetic disease in the patient’s relatives.

We are interested in your opinion on this test. Our study aims to collect the opinion of any person having reached the age of majority, living in France, although these tests are only available abroad. For that we need a few minutes of your time (5 to 10 minutes) to answer to this questionnaire, which is confidential and anonymous.

**Questions:**

**1. Are you aware of this type of test?**

🞏 Yes

🞏 No

🞏 No answer

**2. Would you be in favor of an access to this type of test in France?**

🞏 Yes

🞏 Yes, in case of regulated procedures

🞏 No

If your answer is ‘No’: what are the reasons

🞏 the risk of over medicalization of procreation

🞏 the anxiety that the test may raise

🞏 the consequences on the couple

🞏 a possible questioning of the parental project

🞏 the possible consequences for the child to be born

🞏 my religious concerns

🞏 my ethical or moral convictions (fear of discrimination, of eugenic drift, i.e. discrimination based on genetic criteria)

🞏 other: please precise …………….

**3. In your opinion, what should be the conditions for accessing this test in France?**

🞏 Test accessible to all, with or without a medical prescription

🞏 Test accessible to all, and under medical prescription

🞏 Test accessible according to medical history and under medical prescription

🞏 Test that should not be accessible in France

**4. If you had access to this test while you had a child project, which proposals would your situation best fit?**

🞏 I wish / would have liked to perform this test in case of parental project

🞏 I do not know

🞏 I would not be willing to carry out this test

If your answer is ‘No’: what are the reasons

🞏 the risk of over medicalization of procreation

🞏 the anxiety that the test may raise

🞏 the consequences on the couple

🞏 a possible questioning of the parental project

🞏 a possible consequences on the child to be born

🞏 my religious concerns

🞏 my ethical or moral convictions (fear of a discrimination, of an eugenic drift, i.e. discrimination based on genetic criteria)

🞏 Other: please precise …………….

**5. If you had access to this test while you had a child project, which proposals would your situation best fit?**

(MCQ)

🞏 I will carry out this test only if it is refunded and available in France

🞏 I will carry out this test only if it is available in France even if it's charged

🞏 I will carry out this test only if it is refunded even if it's available only abroad

🞏 I will carry out this test only if it's charged and available abroad

🞏 I will not carry out this test

**6. If this test was available in France, who do you think the test should be proposed to? (MCQ)**

🞏 To any adult wishing to

🞏 To any couple with a parental project

🞏 To any couple having a child with a serious disease

🞏 To a couple having relatives with serious disease

🞏 To any couple in the context of medically assisted procreation

🞏 To nobody

**7. Do you agree with the following proposals?**

| **Proposal** | **Totally agree** | **Somewhat agree** | **Rather disagree** | **Not agree at all** | **Do not know** |
| --- | --- | --- | --- | --- | --- |
| This test may lead to over medicalization of procreation | 🞏 | 🞏 | 🞏 | 🞏 | 🞏 |
| This test may lead to a eugenic drift | 🞏 | 🞏 | 🞏 | 🞏 | 🞏 |
| This test could lead to unnecessary stress for the majority of couples | 🞏 | 🞏 | 🞏 | 🞏 | 🞏 |
| This test could lead to a decrease of the birth rate | 🞏 | 🞏 | 🞏 | 🞏 | 🞏 |
| This test reduces the risk of disability for the offspring | 🞏 | 🞏 | 🞏 | 🞏 | 🞏 |
| This test constitutes a real medical advance | 🞏 | 🞏 | 🞏 | 🞏 | 🞏 |

**8. The financial issue of any new test arises in a systematic way. In your opinion, which of the following is the most acceptable?**

🞏 This test should be systematically proposed and reimbursed by public health insurance

🞏 This test should be available at the request of the patient and reimbursed by public health insurance

🞏 This test should be available at the request of the patient but at his financial burden (estimated price 1000 euros per couple)

🞏 This test should not be available

**9. If the test showed that your couple has a 1/4 risk during each pregnancy to have a child affected by a serious genetic disease, which of the following would most closely match your attitude (in this particular situation)?**

🞏 You would like to know if your fetus is affected or not early during the pregnancy to consider a medical pregnancy termination, depending on the severity of the disease

🞏 You would like to go through an in vitro fertilization step to ensure that you only re implant the embryos without the disease

🞏 You will prefer to schedule a test after birth

🞏 You abandon your parental project or you would consider adopting

🞏 You will not carry out the test during pregnancy or after birth

**10. If you or your partner had a short term parental project, how would you like to be informed about the existence of a test? (MCQ)**

🞏 By the family doctor

🞏 By a gynecologist

🞏 During a consultation of preventive medicine organized by the Social Security at your majority

🞏 Through information provided by the Ministry of Health through the media (social networks, TV, radio)

🞏 Through free information campaigns (TV, radio, internet, magazine ...)

🞏 It does not matter where the information comes from

🞏 Other
