# Supplementary material for: First French study relative to preconception genetic testing: 1500 general population participants’ opinion
Source: Orphanet J Rare Dis. 2021 Mar 12;16:130. doi: 10.1186/s13023-021-01754-z (PMC7955630; doi:10.1186/s13023-021-01754-z)
Supplement: Supplementary file 2 — Additional file 2: Table S1. Summary table of INSEE French demographic data. Table S2. Univariate analysis of questionnaire responses. [file 13023_2021_1754_MOESM2_ESM.docx]

| Characteristics | Year | Category | Percentage |
| --- | --- | --- | --- |
| Gender | 2014 | Women  Men | 52%  48% |
| Ages | 2018 | [18-28]  [28-38]  [38-50]  [50-65]  +65 | 16%  16%  20%  24%  24% |
| Degree | 2014 | Without a degree or without bachelor's degree or high‑shool diploma  Bachelor's degree, or equivalent  Short higher educational studies  Long higher educational studies | 31.4%  40.8%  12%  15.8% |
| % of physicians | 2016 | Physician | 0.3% |

**Table S1. Summary table of INSEE French demographic data**

| Questions | Answer | | | | Number of participants | Percentage |
| --- | --- | --- | --- | --- | --- | --- |
| 1. **Are you aware of this type of test?** | Yes | | | | 452 | 29% |
|  | No | | | | 1114 | 71% |
|  | No answer | | | | 2 | 0.01% |
| 1. **Would you be in favor of access to this type of test in France?** | Yes | | | | 471 | 30% |
|  | Yes, in case of regulated procedures | | | | 955 | 61% |
|  | No | | | | 142 | 9% |
| 1. **In your opinion, what should be the conditions for accessing this test in France?** | Test accessible to all, with or without a medical prescription | | | | 323 | 21% |
|  | Test accessible to all and under medical prescription | | | | 771 | 49% |
|  | Test accessible according to medical history and under medical prescription | | | | 382 | 24% |
|  | Test that should not be accessible in France | | | | 91 | 6% |
| 1. **If you had access to this test while you had a child project, which proposals would your situation best fit?** | I wish / would have liked to perform this test in case of parental project | | | | 893 | 57% |
|  | I do not know | | | | 371 | 24% |
|  | I would not be willing to carry out this test | | | | 304 | 19% |
| 1. **If you had access to this test while you had a child project, which proposals would your situation best fit?**   **(MCQ)** | I will carry out this test only if it is refunded and available in France | | | | 1064 | 68% |
|  | I will carry out this test only if it is available in France even if it's charged | | | | 269 | 17% |
|  | I will carry out this test only if it is refunded even if it's available only abroad | | | | 90 | 6% |
|  | I will carry out this test only if it's charged and available abroad | | | | 37 | 2% |
|  | I will not carry out this test | | | | 276 | 18% |
| 1. **In case in which this test will be available in France, who do you think the test should be proposed to? (MCQ)** | To any adult wishing to | | | | 364 | 23% |
|  | To any couple with a parental project | | | | 821 | 52% |
|  | To any couple having a child with a serious disease | | | | 763 | 49% |
|  | To a couple having relatives with serious disease | | | | 793 | 51% |
|  | To any couple in the context of medically assisted procreation | | | | 385 | 25% |
|  | To no one | | | | 70 | 4% |
|  |  | Totally agree | Somewhat agree | Rather disagree | Not agree at all | Do not know |
| 1. **Do you agree with the following proposals?** | This test may lead to over‑medicalization of procreation | 282 (18%) | 562 (36%) | 390 (25%) | 199 (13%) | 119 (8%) |
|  | This test may lead to a eugenic drift | 388 (25%) | 506 (32%) | 377 (24%) | 207 (13%) | 80 (5%) |
|  | This test could lead to unnecessary stress for the majority of couples | 307 (20%) | 542 (35%) | 404 (26%) | 238 (15%) | 61 (4%) |
|  | This test could lead to a decrease of the birth rate | 98 (6%) | 265 (17%) | 559 (36%) | 478 (31%) | 143 (9%) |
|  | This test reduces the risk of disability for the offspring | 551 (35%) | 703 (45%) | 126 (8%) | 63 (4%) | 109 (7%) |
|  | This test constitutes a real medical advance | 582 (37%) | 680 (43%) | 98 (6%) | 102 (7%) | 96 (6%) |
| 1. **The issue of financing of any new test arises in a systematic way. In your opinion, which of the following is the most acceptable?** | This test should be systematically proposed and reimbursed by public health insurance. | | | | 344 | 22% |
|  | This test should be available at the request of the patient and reimbursed by public health insurance. | | | | 873 | 56% |
|  | This test should be available at the request of the patient but at his financial burden. (estimated price 1000 euros per couple) | | | | 186 | 12% |
|  | This test should not be available. | | | | 143 | 9% |
| 1. **If the test showed that your couple has a 1/4 risk during each pregnancy to have a child affected by a serious genetic disease, which of the following would most closely match your attitude (in this particular situation)?** | You would like to know if your fetus is affected or not early during the pregnancy to consider a medical pregnancy termination, depending on the severity of the disease | | | | 1050 | 68% |
|  | You would like to go through an in vitro fertilization step to ensure that you only re‑implant the embryos without the disease | | | | 194 | 12% |
|  | You will prefer to schedule a test after birth | | | | 100 | 6% |
|  | You abandon your parental project or you would consider adopting | | | | 58 | 4% |
|  | You will not carry out the test during pregnancy or after birth | | | | 152 | 10% |
| 1. **If you or your partner had a short‑term parental project, how would you like to be informed about the existence of a test? (MCQ)** | By the family doctor | | | | 981 | 63% |
|  | By a gynecologist | | | | 1010 | 64% |
|  | During a consultation of preventive medicine organized by the Social Security at your majority | | | | 168 | 11% |
|  | Through information provided by the Ministry of Health through the media (social networks, TV, radio) | | | | 292 | 19% |
|  | Through free information campaigns (TV, radio, internet, magazine ...) | | | | 176 | 11% |
|  | It does not matter where the information comes from | | | | 309 | 20% |
|  | Other | | | | 35 | 2% |

**Table S2. Univariate analysis of questionnaire responses**
